# Experiences of robotic surgery nurses regarding technostress: a qualitative study

**DOI:** 10.1007/s11701-025-02320-6

**Published:** 2025-04-14

**Authors:** Tuğba Sınmaz, Öykü Kara, Ezgi SEYHAN AK, Ayfer Özbaş

**Affiliations:** 1 Nursing Department, Bandirma Onyedi Eylul University Faculty of Health Sciences, 10200 Balikesir, Türkiye; 2 Department of Surgical Nursing, Florence Nightingale Faculty of Nursing, Istanbul University-Cerrahpaşa, Istanbul, Türkiye; 3 Nursing Department, Demiroğlu Science University Florence Nightingale Hospital School of Nursing, Esentepe, Yazarlar Sk. No:17, 34394 İstanbul, Türkiye

**Keywords:** Experiences, Nursing, Robotic surgery, Robotic surgery nursing, Technostress

## Abstract

To qualitatively examine the experiences of robotic surgery nurses regarding technostress. It is thought that determining the impact and importance of technostress on nursing practices and roles that may be caused by robotic surgery technology, which has been widely used, will lead to the development of strategies to prevent or reduce technostress by understanding the challenges and opportunities involved in using new technology. This study was conducted in the operating theatre of a university hospital in Istanbul between May and September 2022. The sample of the study consisted of 11 operating room nurses with at least 6 months of robotic surgery nursing experience who agreed to participate in the study on the dates of the study. The data of the study were collected with a ‘Personal Information Form’ consisting of 14 questions and a ‘Semi-Structured Interview Form’ consisting of 6 questions, which were created in line with the literature and audio-recorded by in-depth interview technique. The majority of the robotic surgery nurses who participated in the study were female and received training on robotic surgery nursing. As a result of the analysis, the themes of the effect of technology on the practices of robotic surgery nurses, education and skill development, work stress and management, team dynamics and cooperation, thoughts about the future of robotic surgery, and suggestions for robotic surgery were obtained. The results of this study revealed that robotic surgery nurses working in high-tech environments are at high risk for technological stress.

## Background

Although the digitalized working environment, which is increasingly shaped by technological advances in professional life, improves performance by enabling faster and more accessible information in numerous areas, as a result of the digitalization of supportive systems and tools in the workplace, it can cause problems related to physical, psychosocial and cognitive dimensions [1]. With the transformation in information and communication technologies, healthcare professionals working in high-tech work environments are exposed to new risks that create stress for organisational, environmental and personal reasons. One of these stress sources is the concept of technostress caused by technology [2].

Technostress was first expressed by Brod in 1984 as ‘a modern maladaptive disease resulting from the inability to encounter new computer technologies in a healthy way and the lack of ability to cope with new technologies’ and can be defined as ‘the feeling of psychological and physical discomfort that occurs when new technologies are used [2]. Technostress is related not only to technology, but also to the changing requirements in many areas, such as the processes and evaluation of learning arising from the use of technology [3]. Technostress is an extremely comprehensive phenomenon; “techno-overload”, which occurs when technology forces individuals to work faster; “techno-complexity”, which is related to users' perception that their technological skills are inadequate; “techno-insecurity”, which involves users feeling under the threat of losing their jobs due to technology or others with better technical knowledge; “techno-uncertainty”, which is related to the constant changes and developments that occur due to technology; and “techno-invasion”, which is related to the blurring of work and home life due to technology [1, 4].

Technostress is a global phenomenon, and its incidence is reported to be 25–39% in countries such as the United States, China and Australia. Technostress occurs among healthcare workers because of insufficient experience, uncertainty about device interruptions requiring repair, loss of control, etc. According to Bloom, some of the main causes of technostress are insufficient experience and skills, performance anxiety, lack of training, organisational factors, an insufficient number of personnel, and rapid changes in information and technology [5]. Although studies examining technostress in healthcare settings are limited [6], Mahdian et al. (2017) examined technostress in nursing and reported that technostress in nurses was moderate. Abuatiq (2015) reported that technostress has a negative effect on performance by shifting an individual's stress, which is compatible with the job, to an internal distress state [7].

Rapid developments in the area of surgery, including high-technological work environments using the latest technology and changes in structures and processes, require innovations, and nurses working in these areas face a greater risk of technostress due to a number of technostressors, such as the responsibility of nurses working in these areas to efficiently integrate continuously occurring changes and new technological resources and equipment into the work environment and to operate new devices [8]. Since robotic surgical interventions require complex medical interventions, a continuous education process is needed for nurses to achieve the desired competence to improve patient safety [2]. The first step in training is to learn the equipment and terminology related to robotic surgery, and the second step is to set up the system. The role of the robotic surgery nurse in the preoperative period is to prepare and control the system, position the patient and ensure the safety of the team. The responsibilities of the nurse during the operation include assisting the surgeon, paying attention to the rules of asepsis by distinguishing the sterile and nonsterile parts of the robot, placing the robot in the body, and reading and reporting the data from the videoscopic screen accurately and quickly. In robotic surgery, which is an area where teamwork significantly affects success, the responsibility of the nurse is to establish the system and ensure its continuity throughout the procedure [9, 10].

New ideas and more advanced models are designed every day for robotic surgery. As robotic surgery technology develops, the dimensions decrease, and functionality increases. It is thought that the use of robotic systems with high technology and the necessity of managing the relationship between nurses and the team may lead to the development of technostress in nurses. Determining the effect and importance of technostress that may be caused by robotic surgery technology, which has been widely used, on nursing practices and roles will help to develop strategies to prevent or reduce technostress by understanding the challenges and opportunities involved in using new technology and how to cope with them as health technology develops. However, there are no studies in the literature concerning technostress experiences, which may be caused by the use of advanced technology in robotic surgery teams, including robotic surgery nurses. This study was conducted to examine the experiences of robotic surgery nurses regarding technostress.

## Methods

### Aim and design

This study aimed to qualitatively examine robotic surgery nurses' experiences of technostress. A descriptive phenomenological research design, a qualitative research method, was used in this study. Descriptive phenomenology is used to understand individuals’ experiences, the meaning of these experiences and their thoughts about the phenomenon [11]. The reason for choosing this approach in this study was to reveal the experiences of robotic surgery nurses regarding technostress from their own perspectives and to understand their feelings, thoughts and experiences regarding the situation. The study is reported according to the consolidated criteria for reporting qualitative research (COREQ) [12] (Appendix 1).

### Sample and population

The population of the study consisted of nurses on the robotic surgery team of a university hospital between May 2022 and September 2022, and the sample consisted of operating theatre nurses who worked as robotic surgery nurses for at least 6 months and agreed to participate. In this study, a purposive sampling technique was used to collect data from individuals.

In qualitative research, when the content obtained from the individuals participating in the interview begins to repeat itself and there is no new finding to be added, it is considered that data saturation has been reached and the sample size is sufficient [13, 14]. The sample size was determined according to the data saturation point, a method commonly used in qualitative research. When the themes and categories obtained from the participants started to be repeated, data saturation was reached. This study was completed with 11 nurses.

### Data collection tools

The research data were collected with a personal information form and a semistructured interview form, and a voice recorder was used during the interviews.

#### Personal information form

A form consisting of 14 questions created by researchers through a review of the literature was used [2, 10, 15]. In the last 3 questions, the level of trust in robotic systems, the level of complexity of robotic systems, and the level of stress related to technological problems experienced during robotic surgery were evaluated on the basis of subjective reporting with a visual analog scale. A visual analog scale (VAS) is used to express numerical values that cannot be measured numerically. The measurement is made by writing the parameter to be evaluated on the two ends of a 10 cm line and marking the place where the person's condition is appropriate on this line. It is a safe and easy-to-use tool that does not have any language, is easy to apply, has proven itself for a long time and is accepted worldwide [16]. In this study, the VAS was used in a 10 cm horizontal format.

#### Semistructured interview form

This form, developed by researchers after a literature review, consists of 6 open-ended questions [7, 15, 17]. The final version of the questions in the questionnaire was determined after expert opinion and pilot application. The questions in the questionnaire are listed below:

- How does technology affect your work life/profession?
- What difficulties did you encounter in adapting to the robotic surgery team and systems?
- How has technology affected your role/duties as operating room nurses?
- Do robotic surgery systems expose you to stress? If yes, ‘Do you think your stress as a robotic surgery nurse is due to the nature of your profession or technological systems?’
- What kind of physical and behavioral negativity does the stress caused by the possibility of encountering technological problems during robotic surgery interventions cause you to experience?
- What kind of developments do you foresee in robotic surgery nursing in the future?

### Implementation of the study

Before data collection, the study was introduced to the robotic surgery operating room nurses, and the nurses were invited to participate in the study. The conditions for participation in the study and the contact information of the researchers were shared with the relevant nurses. Since they were active employees, special appointments were made, and data were collected via face-to-face individual in-depth interviews. Before the interviews began, a face-to-face pilot interview was conducted with two participants who were not included in the sample. In line with these interviews, the interview process and questions were reorganized. After the nurses were informed about the purpose and method of the study, the telephone numbers of the nurses who volunteered to participate in the study were obtained, and an interview was planned for convenience. Verbal and written permission was obtained from the nurses who agreed to participate in the study at the beginning of the interview. In this study, a personal information form consisting of 14 questions about the descriptive characteristics of the nurses and a semistructured interview form were used to collect data through face-to-face individual in-depth interviews. The interviews were conducted in a quiet room in the operating theatre suitable for mutual interviews. In line with ethical approaches, the real names and surnames of the participants were not used, and each of them was numbered according to the order of the interviews. The interviewer and the participant were alone during the interview. Each interview lasted approximately 45 minutes and was recorded. One of the researchers who conducted the interviews had a doctorate degree and the other had a specialist degree. Two of the researchers had a ‘qualitative research course participation certificate’, and three researchers were also involved in the ‘robotic surgery nursing certificate programme’ as trainers.

### Ethical approach

Before starting the study, ethics committee permission (Date: 25.05.2022/Number: 392002) and permission were obtained from the institution where the study would be conducted (Date: 16.06.2022/Number: 408848). Since the use of human phenomenon in the research requires the protection of individual rights, verbal and written 'informed consent' was obtained from the individuals participating in the research. The confidentiality of the participants was protected at all stages of the study, and no practice or behaviour contrary to ethical values was performed. The participants were informed that all the information received would be kept by the researchers and that the answers would remain confidential and would be used only for scientific purposes. Owing to concerns about the protection of privacy, the real names and other personal information of the interviewees were not used in this study, and for this purpose, all the data were anonymized by numbering them according to the interview order. A voice recorder was used in the study. No one other than the researchers listened to the recordings. All records and permissions belonging to the research are protected with digital passwords and will be destroyed in accordance with the law of destruction at the end of 5 years after the publication process is completed.

### Data analysis

The voice recordings were transcribed by the researchers and checked for consistency. In the analysis of the data, the MAXQDA 20.0 software and Colaizzi’s (1978) phenomenological analysis steps were used [18]. The data were coded independently by the researchers by developing a code list, and a consensus was reached on the codes. To ensure intercoder reliability, the codes were compared, and inconsistencies were resolved by consensus. Themes were extracted from the data. The transcripts were read several times by the researchers, and short notes were taken. Important statements were selected and reviewed, and those with common meanings were grouped. These meanings were categorized into themes, categories and codes, and the data were reported within the framework of the themes. The results were combined with life experiences. The conceptual structure of the analysed phenomenon was revealed. The findings were then verified by the participants. The results obtained were checked by a field expert.

## Results

### Descriptive characteristics

Descriptive characteristics are given in Table 1.

**Table 1:** Descriptive characteristics (N = 11)

Variables	Count (n)	Percent (%)
Age (x̄ ± SD: 31.45 ± 6.251, Min–max:25–44)		
Duration of work in the profession (year) (x̄ ± SD: 9.73 ± 6.71, Min–max:3–25)		
Duration of work in the operating room (year) (x̄ ± SD: 9 ± 6.723, Min–max:3–25)		
Duration of work in the organization (year) (x̄ ± SD: 7.91 ± 4.826, Min–max:2–17)		
Duration of work as a robotic surgery nurse (year) (x̄ ± SD: 1.36 ± 0.674, Min–max:1–3)		
Sex		
Female	8	72.7
Male	3	27.3
Marital status		
Single	2	18.2
Married	9	81.8
Educational status		
University	10	90.9
Postgraduate	1	9.1
Robotic surgery branches worked in*		
General surgery	9	25.7
Urology	11	31.4
Thoracic surgery	5	14.3
Obstetrics and gynecology	7	20
Pediatric surgery	2	5.7
Plastic surgery	1	2.9
Status of training on robotic surgery		
Yes	9	81.8
No	2	18.2
Institution received training		
Another university hospital	4	44.4
Organization worked for	1	11.2
Company	4	44.4
Thinking that the current tasks in the robotic surgery team are defined correctly		
Yes	4	36.4
No	3	27.2
Not sure	4	36.4
Level of trust in robotic surgical systems** (x̄ ± SD: 7.82 ± 0.982, Min–max:6-9)		
Level of complexity of robotic surgical systems** (x̄ ± SD: 3.91 ± 1.921, Min–max:2–8)		
Stress level related to technological problems experienced during surgery** (x̄ ± SD: 4.82 ± 2.75, Min–max:1–9)		

*More than one answer was given

**In the range of minimum 0 to maximum 10

### Themes, categories and codes obtained from participants' experiences

The experiences of the participants were grouped into 6 themes: the effect of technology on the practices of robotic surgery nurses, education and skill development, work stress and management, team dynamics and cooperation, thoughts on the future of robotic surgery, and suggestions for robotic surgery (Table 2).

**Table 2:** Themes, Categories and Codes of the Study

Theme	Category	Code
1. The effect of technology on the practices of robotic surgery nurses	Effective use of technological tools	Increasing work efficiency reduction of workload
	Change in working processes	Automation of workflows
		Adaptation to new work models
	Technological problems and difficulties	Rapid response to faults
		Improving technical support and maintenance services
2. Training and skills development	Continuing education requirement	Continuing education for areas of expertise
	Implementation difficulties	Reinforcement of theoretical knowledge with practices
		Development of clinical skills
3. Work stress and management	Stress sources	Workload and performance stress
		Technological problems and interruptions
	Strategies for coping with stress	Stress management techniques
		Improvement of the working environment
4. Team dynamics and collaboration	Team communication	Establishing open and effective communication channels
		Developing feedback and appreciation culture
	Adaptation problems and solutions	Strengthening conflict resolution mechanisms
		Team creation and team spirit development activities
5. Future Considerations for Robotic Surgery	Simplification of robotic surgery	Increasing ease of use
	Use and spread of robotic surgery in more areas	Dissemination in public and private sector
		Increasing accessibility and cost effectiveness
6. Recommendations for robotic surgery	Use of more convenient robots	More ergonomic design
		Better response times
		Increased automation level
	Requirement for more experience in robotic surgery	Education programmes
		Providing experience in real cases
		Mentoring and counseling
		Continuous professional development
	Standardization of trainings provided	Development of certification standards
		Keeping the training curriculum up to date
	Increasing technology literacy	Integration into the undergraduate curriculum
		Receiving support from different disciplines
	Defining the Role Description of Robotic Surgery Nursing	Clear definition of roles and responsibilities
		Clarifying professional roles

### Theme 1: impact of technology on robotic surgery nurses' practices

The participants evaluated the effects of technology on a wide range of nursing practices. These impacts were categorized as the effective use of technological tools, changes in work processes, technological problems and challenges.

#### Effective use of technological tools

The majority of the participants stated that the use of technological tools increased work efficiency by reducing workload, whereas others stated that it increased workload. They also emphasized that the use of technological devices in surgical procedures provides safe results and that having the necessary knowledge about the use of technological devices makes them feel safer.

‘I mean, I think it makes our work faster and easier. However, first, we need to have good information about the situation. When we have good information, I feel safe. If I have very little information, I get truly anxious when something goes wrong.’ (P2)

‘Compared with open surgery, you are more comfortable, the case can be monitored, more controllable, so you can see if there is bleeding or not? You can follow the next movement; accordingly, our job is of course a little easier.’ (P3)

‘Although it seems to increase our workload from time to time, I think that it enables us to achieve safer results by increasing the quality and safety of work in the long term. When used correctly, I think that productivity in terms of time, money and workforce will increase.’ (P1)

#### Changes in working processes

The importance of the process of adaptation to new business models was also emphasized by the participants, who stated that owing to technological tools, they expended less physical effort and were in the role of passive observers, as many procedures that were previously performed manually became automated; however, especially in areas where technology is used intensively, such as robotic surgery, the importance of the adaptation process to new business models was also emphasized by the participants, as the preparation and finalization processes are a cognitively demanding area, as they require more attention and care than standard surgical procedures do.

‘Technology did not directly affect my work but increased my workload, definitely not a standard desk nursing.... Although not physically, robotic surgery is more tiring than others in terms of stress load and as a department that requires attention.’ (P10)

‘It affected our role and tasks in a good way. It reduced our workload, and we started to do things in more practical ways.’ (P4)

‘... in fact, as long as there is technology, our work becomes easier, but it does not push nursing into the background a little bit. For example, the nursing we do in an open surgery and the nursing we do in robotic surgery are not much the same. Therefore, it is positive for the patient, but it is a little less satisfying for us.’ (P8)

#### Technological problems and difficulties

Participants stated that in addition to the conveniences brought by technology, the complexity of technology can make their work difficult, especially in emergencies or unexpected failures.

‘...Of course, I am aware that there may be crises and that it is necessary to be alert. Because I was afraid that the arms of the robot would be separated from the patient in case of the slightest bleeding.’ (P7)

‘Well, sometimes it can be complicated because what we are working with is a machine, so when there are things that we cannot solve, there are situations where we have to wait a little bit and prolong the process.’ (P4)

‘Sometimes, the device does not accept the arm during the docking phase; we have problems at that time, but we have not experienced any major complications.’ (P9)

‘Maintaining sterilization can be difficult as follows: our camera optics are very big, and it is very difficult to protect it while attaching it to the robot.’ (P11)

### Theme 2: education and skills development

Within the scope of the second theme, categories related to the need for continuing education and implementation difficulties were obtained from the statements of the participants.

#### Continuing education requirement

The participants stated that having the necessary knowledge for the effective use of technological tools made them feel more confident and that training, especially in high-tech areas such as robotic surgery, is a constant requirement for the development of technological skills and access to up-to-date information.

‘It creates the need to revise knowledge and experiences with technology.’ (P5)

‘When the first robot came, they started with me, I had already started without any training. The robot came very fast, there was actually no time left for training... I was very stressed that day because I was very afraid of doing something wrong.’ (P10)

‘...since they explain everything systematically in the training, when you come here, this arm is broken, I can do this, so you need to be trained for this. I think so. You become more self-controlled, you become more confident.’ (P8)

#### Implementation difficulties

To overcome the difficulties experienced during the use of these technologies, the statements of the participants emphasize the importance of providing training in a way that will enable the development of clinical skills by reinforcing the theoretical knowledge with practical applications and eliminating the lack of communication and coordination.

‘I think surgeons should definitely undergo a certificate programme like the training given to us, because these are the tools of the next period, so everyone should receive training. This kind of training should be prepared for doctors as well. ...when you go there with training, you approach it more consciously.’ (P2)

‘Therefore, there may be a communication problem. Let me tell you as much as I have seen from outsiders. Since they are newcomers, they take the console directly, for example, without receiving any feedback.’ (P6)

‘Actually, I think there is more risk in robotics. Simply put, when our assistant is trying to insert something through the auxiliary port, he cannot see the port and tilts his head, whereas the man at the console cannot see the table, so he may make a different movement and hit his head. In this regard, as a nurse, we are constantly warning ‘do not bend too much, do not get too close, it might hit your face’ because there are sudden movements. I mean, we cannot predict this because we cannot look at the teacher and understand how the hand gesture is. Therefore, we have to be slightly more cautious.’ (P8)

### Theme three: work stress and management

#### Sources of stress

The participants stated that workload and performance pressure, technological malfunctions and interruptions constitute the majority of sources of stress at work. In addition, the cost of technological devices and their maintenance are also important sources of stress.

“If I have very little knowledge, I get truly anxious when there is a problem. Or when it breaks down, there are problems such as not having a backup; in other words, we have limited resources. Since technological devices are expensive, we have only one of them in our institution. When it breaks down, you are left alone, so I feel safe when there is a backup.” (P2)

“… there is something called a red alert; when that happens, we experience great stress because it is an irreversible error. In other words, completely shutting down the system is an alarm that we fear because there are problems such as removing the devices inside the patient.” (P6)

“There were a few bleeding events while on the table; the biggest complication was that we had very little time to separate the robot from the patient during bleeding and switch to open surgery. Panic sets in, you have to be quick at that moment.” (P10)

“… there is hesitation when you have something you don’t know about different technological devices. You have to keep it in your hand, where will I open it, where will I close it. Therefore, of course, you have great stresses. There is a machine in front of you that you do not know, you will use it, what will the surgeon want from me, where should I press, these happened. It is the same with the robot, for example, we placed the arms, which arm should I give first, this causes a lot of stress." (P8)

"Let me put it this way, I sweat. I sweat excessively, I get stressed, I get a fever, etc., but it passes later." (P11)

#### Strategies for coping with stress

The participants emphasized the stress management techniques they use when faced with stress and the need to improve the work environment in managing workplace stress.

“…At first, there was a fear of the unknown, but since the company’s technical support was always with us, I never went into the psychosis of what to do now, we knew we had to call them, they were always with us, we would only feel a momentary tension for a few seconds, and then it would resolve anyway. In other words, this was not about us, but about the company, they always gave us that confidence.” (P1)

“I don’t think there is a situation like I am very stressed due to robotic surgery; in contrast, I go to robotic surgery because it is a calm, slow system where everything is clear step by step.” (P4)

“In other words, all the previous problems with the robot, etc., were explained to us. There was also the issue of connecting remotely from the internet, there were their error codes, etc. When we contacted them with error codes other than those we could solve ourselves, the company directed us anyway. There are already people here… The lady looks after us; we could always reach her.” (P5)

### Theme four: team dynamics and collaboration

#### Team communication

The statements of the participants reveal in detail the difficulties of communication within the team and the importance of establishing open and effective communication channels and developing a culture of feedback and appreciation in overcoming these difficulties.

“Both within the profession and among all healthcare personnel, namely, the manager, sometimes resisted me because I made the decision, even if it was the right one. There is always a hierarchy; I encountered resistance at this point among surgical nurses from time to time. However, at the end of the day, when they found the truth and saw my effort, they were all convinced.” (P1)

“…Many of them were disturbed by the fact that I was giving the appointments. Then, they were disturbed by the fact that I was setting up the robot or they were disturbed by me saying stop. However, they did not know, I had the most trouble with these, I had no problems with the system itself. I encountered individual resistance in terms of creating the infrastructure, ensuring its continuity and convincing the teams.” (P11)

“…Normally, our job was not a job that was carried home; I would leave the table, and my shift would be over. There was no situation where I would do work at home, but my phones started to stop ringing… I experienced the stress of the team because they trusted me because there were situations I had not encountered.” (P10)

#### Adaptation problems and solutions

A lack of clear job definitions and boundaries often causes disagreements and adaptation problems within the team and can become even more complicated, especially with the integration of new technologies and changes in business processes.

“When we look at the institution as a whole, since we were a new department, we had a lot of trouble in terms of job descriptions and taking responsibility at first. We established a department from scratch, I had to think about all the details and take ownership of it. I had trouble convincing people of this. Then, we entered into a conflict of ego and knowledge. In other words, I encountered a group of surgeons who did not accept a nurse giving them training and making decisions on their behalf. I was criticized by my own colleagues.” (P1)

“Sometimes seeing that the case duration will be very long can bring down my mood. I know that I would want to work with a better team, a team where I would feel better.” (P2)

“…we never had any problems in terms of communication. Because we know the case, we have been working on this case for years, we know what to use, we know what to do when there are any complications, so we do not have any problems with our surgeons in this regard. However, we had a lack of communication with the units we worked with less.” (P8)

### Theme five: thoughts on the future of robotic surgery

#### Simplification of robotic surgery

A participant's statement emphasized the potential of simplifying procedures in robotic surgery and the importance of steps to be taken in this direction. He suggested that increasing the ease of use of robotic systems can make it possible to complete operations with fewer personnel faster and with fewer errors and that strong cooperation and communication skills among team members are also highly important in this process.

“For example, we heard that abroad, a doctor and a nurse can perform a case alone, without needing anyone. This can actually be done in the future, but both parties need to know each other very well in this regard.” (P2)

#### Use and spread of robotic surgery in increased areas

The participants expressed their views that robotic surgery would be used in more areas and that the integration of this technology into healthcare services would deepen.

“…in the future, robots will become such that I think surgeons will do this job from home. In other words, they will perform operations with virtual glasses that children play with and put on their eyes.” (P8)

“…I think robotic surgery will be in every center, I mean, it seems very expensive right now, but I think these will become very cheap in the future. In other words, just as not every person had a telephone or a television in the past, even people who are not well off have television now. It will come to that point. In other words, I think there will be a robotic surgery console in every hospital.” (P9)

### Theme six: recommendations for robotic surgery

#### Use of more useful robots

The participants stated that more ergonomic designs using wireless systems can reduce the physical restrictions encountered during operations and that robotic surgery systems can also simplify maintenance and repair processes. They emphasized that robotic surgery equipment has faster response times and increased automation levels, allowing surgical procedures to be performed more comfortably and without interruption.

“We can switch to a wireless system because it is always on the ground; we are careful not to step on it; these may change. It would be better if there was a wireless system.” (P4)

“It can be difficult to maintain sterilization; our camera optic is very large, we use SI, it is very large, and it is a little difficult to protect it when attaching it to the robot.” (P9)

“…sometimes the arm does not accept the device during the docking phase in the arms, we have problems at that time.” (P7)

#### Requirement for more experience in robotic surgery

The participants stated that when they do not have enough experience, they feel less confident during robotic surgery, which can increase their stress levels, increase surgical errors and reduce patient safety.

“Experience also comes into play here. If I had slightly more experience, I probably wouldn’t feel so confident. Right now, I am a little stressed because I don’t feel that experienced with the robot. The master‒apprentice relationship should probably be slightly more.” (P2)

“I think it makes our job easier. However, we need to have good knowledge about that situation beforehand. I feel safe when we have good knowledge. If I have very little knowledge, I become truly anxious when there is a problem. (P11)

“…if the teams know something, I think we all have an idea right now, at the beginning they didn’t have an idea either, I had very little idea, we didn’t know what to do. We were more worried then, but now it has less.” (P1)

#### Standardization of training provided

Some of the participants stated that standardized, up-to-date and comprehensive training programs for robotic surgery can ensure the quality of training; ensure that all healthcare professionals, from nurses to surgeons, have the same level of knowledge and skills; adapt to rapidly and continuously developing technology; and thus reduce possible errors by using robotic systems effectively and safely.

“I definitely think assistant surgeons should also go through a certification program like the training we received because these are the tools of the future, so everyone needs to receive training. I think it is something that already exists in our hospital and will be even more so in the future. Such training should also be prepared for doctors. When you go there after receiving training, you approach the place more consciously.” (P2)

“…since they explain everything systematically in training, I can do it when my arm breaks when I come here, so I need to receive training for this. You become more self-controlled and confident.” (P9)

#### Increasing technological literacy

Some of the participants emphasized that technology education should be integrated into the curriculum of undergraduate nursing education as a basic component to ensure that health professionals use technological tools more effectively and reduce surgical errors and that support can be received from different disciplines in this process.

“I think technology literacy is definitely something that should be included in the nursing curriculum now. At least you need to know the meaning of an on/off button in several languages.” (P1)

“…In other words, by being exposed to it at least once by giving examples, you can also receive support from an engineer in the existing system; support can be received from informaticians, but technology literacy should be within nursing education.” (P10)

#### Defining the role description of robotic surgery nursing

The participants stated that the vagueness of the job descriptions and responsibilities of robotic surgical nursing caused disagreements and confusion in work processes both within the institution and among professionals.

“The job description, the lack of defined job boundaries, and the fact that it was not clear who would use this technical part caused a confusion of concepts and job description confusion from time to time.” (P1)

“I don’t know that robotic surgical nursing has a job description; it is the same as normal surgical nursing. Should our job description be developed? I think it should be developed. We do everything as long as it benefits the patient. So I don’t know if this makes sense either. This will just be our assurance.” (P8)

“Therefore, your job description is actually clear, but of course, these things can change during the case. Sometimes, for example, it becomes the assistant surgeon’s job, and you have to get support.” (P9)

## Discussion

Technological applications like robotic surgery reduce bleeding risk and allow more controlled interventions [9, 19]. The study found high confidence in robotic surgery systems, leading to safer patient outcomes and making nurses' jobs easier and safer. Schuessler et al. (2019) and Randell et al. (2019) reported that nurses preferred robotic surgery over laparoscopic surgery and saw it as a chance for professional development [20, 21]. However, complex devices, malfunctions, and lack of experience can lead to "techno-complexity," a form of technostress linked to inadequate technology skills. High costs and limited resources can also make nurses' jobs more stressful and prolong work processes. The robotic surgery system's complexity was found to be low in this study, but rapid integration of new technology without proper training can hinder effective use. Additionally, maintenance and sterilization of high-tech devices can increase nurses' workload and stress, raising the risk of errors.

The integration of technology in robotic surgery is changing the roles of nurses, sometimes making them more passive observers [19]. Redondo-Saenz et al. (2023) noted that robotic technology can distract nurses from patient interaction, potentially impacting job satisfaction, especially for those who prefer hands-on roles [22]. Technologically skilled nurses adapt quickly, but older nurses may face more challenges, depending on their ability to adapt [23]. This can lead to "techno-insecurity," where nurses fear losing their jobs to those with better tech skills. One participant mentioned that his expertise in robotic surgery led to constant calls after his shift, illustrating "techno-invasion," a form of technostress.

Communication challenges hinder technological integration, especially in robotic surgery, where the surgeon’s distance from the patient can affect team coordination [24]. Using wireless headsets improves communication quality [25]. Nurses must effectively convey challenges with technology to receive timely support [26].

Continuous and systematic training in high-tech fields like robotic surgery is crucial for developing technological skills and keeping healthcare personnel updated [15, 27]. Similar to Uslu et al. (2019), participants in this study noted that unclear job descriptions and responsibilities, especially with new technologies, create confusion and disrupt workflows [10]. This leads to "techno uncertainty," a form of technostress linked to continuous technological changes. To address this, training programs should clarify job roles and provide technical knowledge. Schuessler et al. (2019) emphasized the need for standardized education and procedural guidelines for nurses to ensure efficiency. While most participants received robotic surgery training, it varied by institution, and those without institutional support had slower learning experiences [20]. Uslu et al. (2019) found that nurses preferred professional training over the master-apprentice approach and wanted ongoing training [10]. Continuous education is key to updating skills, addressing technological challenges, and ensuring safer healthcare services.

Technology integration in healthcare presents challenges, particularly due to limited training. Nurses express a need for updated education on robotic-assisted surgery, including console operation and emergency management [15]. Communication and coordination issues further complicate technology use [28], as robotic-assisted surgery reduces nonverbal and verbal communication [29]. Effective training must address both technical and teamwork skills. Adapting to new technologies quickly is crucial, as inadequate training is a leading cause of malfunctions and stress [20, 30]. Additionally, concerns over the cost and protection of devices add pressure on healthcare professionals [19]. Training should therefore emphasize proper use, maintenance, and care to alleviate these challenges.

Nurses face moderate stress when using technology, particularly during robotic surgery. A study measuring stress levels (VAS 0–10) in 114 nurses found that 40% experienced stress due to the high cost and fear of damaging equipment [30]. Device complexity, potential malfunctions, and emergency response challenges add to their burden. Managing multiple systems increases workload and mental fatigue, while financial concerns over maintenance and damage further heighten stress [9, 15]. Insufficient training and adaptation processes create uncertainty, making nurses feel inadequate and fearful of errors. Team dynamics also contribute to stress, as varying skill levels can lead to communication gaps and conflicts [29]. Effective stress management training can improve job satisfaction, yet its absence limits nurses' ability to cope with these challenges.

With the integration of technology, stress management in nursing is increasingly important. Nurses use various coping strategies that impact their job performance. Technical issues, especially robotic device malfunctions, are major stressors, worsened by a lack of trained support [15]. Accessible technical support helps resolve issues quickly, reducing stress. Effective team communication is also crucial, enabling rapid decision-making in urgent situations and improving both patient care and staff well-being. Well-coordinated teams manage challenges more efficiently, minimizing errors and stress.

Unclear team hierarchy can cause communication issues and resistance to managerial decisions, even when correct. Supportive leadership is key to resolving conflicts and fostering trust. Senior management backing helps overcome resistance and ease transitions. Randell et al. (2019) highlight the importance of surgical team support, experienced nurses, and strong leadership in robotic surgery [21]. Clearly defining robotic nursing roles enhances safety, competence, and patient care quality.

Inexperienced healthcare professionals feel less confident during robotic surgery, increasing stress and the risk of errors. Team-based training enhances safety and reduces preparation time [21]. Standardized robotic surgery training ensures consistent knowledge across roles [31] and certification programs help maintain quality. Standardization also improves collaboration and ensures uniform service across healthcare institutions.

Technological literacy is crucial in robotic surgery, helping healthcare professionals navigate challenges. A study [32] found 84.3% of nursing students were interested in technology, with 73% having some knowledge of robotic surgery, mainly accessed online. However, another study reported 67.5% lacked such knowledge [33]. Integrating technology literacy into nursing education enhances both technical competence and ethical/legal awareness, improving decision-making and efficiency in robotic surgery.

## Limitations

The study was conducted with robotic surgery nurses working in a single center. These research results only reflect the thoughts and experiences of the robotic surgery nurses who participated in the study.

## Conclusions

In conclusion, although the integration of technology provides significant benefits to the nursing profession, this process involves various sources of stress. For nurses to cope with these stresses, continuous education, effective stress management techniques, and the appreciation of everyone's ideas within the team, encouraging everyone's participation, and the development of solid team dynamics that include a culture of appreciation are necessary. Solving intrateam adaptation problems requires a combination of individual and institutional efforts. Such support will enable nurses to use technology more effectively, thus both improving the quality of patient care and increasing nurses' job satisfaction.

## Implications for practice

The benefits provided by the increasing use of technology in many areas, as well as the negative consequences related to stress it causes, are being investigated in many areas, but technostress, which is a biopsychosocial risk factor that nurses working in robotic surgery surgeries, one of the environments where high technology is most frequently used, are exposed to, is overlooked and not sufficiently investigated. This study is expected to be effective in developing coping strategies to prevent or reduce technostress by providing an understanding of the technostress experienced by robotic surgery nurses.

## Data Availability

No datasets were generated or analysed during the current study.
